# Genomic Characterization of *Listeria monocytogenes* and Other *Listeria* Species Isolated from Sea Turtles

**DOI:** 10.3390/microorganisms12040817

**Published:** 2024-04-18

**Authors:** Ludovica Di Renzo, Maria Elisabetta De Angelis, Marina Torresi, Giulia Mariani, Federica Pizzurro, Luana Fiorella Mincarelli, Emanuele Esposito, Maria Oliviero, Doriana Iaccarino, Fabio Di Nocera, Gianluigi Paduano, Giuseppe Lucifora, Cesare Cammà, Nicola Ferri, Francesco Pomilio

**Affiliations:** 1 Istituto Zooprofilattico Sperimentale dell’Abruzzo e del Molise “Giuseppe Caporale”, Via Campo Boario, 64100 Teramo, Italy; l.direnzo@izs.it (L.D.R.); m.torresi@izs.it (M.T.); g.mariani@izs.it (G.M.); f.pizzurro@izs.it (F.P.); l.mincarelli@izs.it (L.F.M.); c.camma@izs.it (C.C.); n.ferri@izs.it (N.F.); f.pomilio@izs.it (F.P.); 2 Centro Studi Cetacei, 65125 Pescara, Italy; 3 Faculty of Bioscience and Agro-Food and Environmental Technology, University of Teramo, 64100 Teramo, Italy; 4 Istituto Zooprofilattico Sperimentale del Mezzogiorno, 80055 Portici, Italy; emanuele.esposito@izsmportici.it (E.E.); doriana.iaccarino@izsmportici.it (D.I.); fabio.dinocera@izsmportici.it (F.D.N.); giuseppe.lucifora@izsmportici.it (G.L.); 5 Azienda Sanitaria Locale Salerno, 84124 Salerno, Italy; g.paduano@aslsalerno.it

**Keywords:** *Listeria monocytogenes*, *Listeria* spp., WGS, marine environment, sea turtle

## Abstract

*Listeria monocytogenes* is a ubiquitous pathogen found both in the environment and food. It can cause listeriosis in a wide range of animals as well as in humans. Investigations on presence, spread and virulence are still limited to terrestrial and human environments. Embracing the One Health Approach, investigating the presence and spread of *L. monocytogenes* in marine ecosystems and among wildlife, would provide us with useful information for human health. This study investigated the presence of *L. monocytogenes* and *Listeria* spp. in two species of sea turtles common in the Mediterranean Sea (*Caretta caretta* and *Chelonia mydas*). A total of one hundred and sixty-four carcasses of sea turtles (*C. caretta n* = 161 and *C. mydas n* = 3) stranded along the Abruzzo, Molise, Campania, and Calabria coasts, were collected. Brain and fecal samples were taken, enriched, and cultured for the detection of *Listeria* spp. From the specimens collected, strains of *L. monocytogenes* (brain *n* = 1, brain and feces *n* = 1, multiorgan *n* = 1 and feces *n* = 1), *L. innocua* (feces *n* = 1 and brain *n* = 1), and *L. ivanovii* (brain *n* = 1) were isolated. Typical colonies were isolated for Whole Genome Sequencing (WGS). Virulence genes, disinfectants/metal resistance, and antimicrobial resistance were also investigated. *L. monocytogenes*, *L. innocua,* and *L. ivanovii* were detected in *C. caretta*, whilst only *L. monocytogenes* and *L. innocua* in *C. mydas*. Notable among the results is the lack of significant differences in gene distribution between human and sea turtle strains. Furthermore, potentially pathogenic strains of *L. monocytogenes* were found in sea turtles.

## 1. Introduction

Marine ecosystems are considered among the most economically and ecologically valuable systems worldwide [1], nonetheless, they are vulnerable to the increasing anthropogenic disturbances [2]. Therefore, investigations are of paramount importance to monitor and preserve the balance of marine ecosystems through sentinel species, whose health status reflects the status of the sea [3,4,5]. Like marine mammals and other marine vertebrates, sea turtles are long-lived organisms, widely distributed from benthic neritic areas and characterized by habitat fidelity [6]. The loggerhead sea turtle (*Caretta caretta—C. caretta*) and the green turtle (*Chelonia mydas*—*C. mydas*) are two of three species present in the Mediterranean basin. The third one, *Dermochelys coriacea* (*D. coriacea*), is rarely spotted in these waters. While the loggerhead sea turtle is very common in the Mediterranean area [7,8,9], the green turtle uses only the eastern part as a nesting area and is rarely spotted on the Italian coasts [10,11]. All of them are included in the Red List of the World Conservation Union (IUNC) as endangered (*C. mydas*) and vulnerable (*C. caretta* and *D. coriacea*) species. Concerning the Mediterranean subpopulation, *C. caretta* has been recently downlisted to “least concern” (LC) according to the Italian IUCN Red List [12]. However, this improvement is strictly dependent on conservation measures. Due to their opportunistic feeding behavior associated with their wide distribution, they bioaccumulate contaminants, toxins, and pathogens [13] through their diet. In particular, the loggerhead sea turtle *C. caretta* feeds mainly on benthic organisms [9,14], therefore can be considered a valuable sentinel especially for nearshore environments [15]. The EU Marine Strategy Framework Directive (MSFD) has chosen the loggerhead turtle as a bio-indicator of marine litter in the Mediterranean Sea. Moreover, they have been adopted as bio-indicators of antimicrobial-resistant bacteria in coastal environments [16], as well as in fibropapillomatosis epidemiology [17].

Listeriosis is a disease that can be transmitted to humans mainly by consumption of food contaminated by *Listeria monocytogenes* (*L. monocytogenes*, *Lm*). It is of high concern for food safety and public health, as it can cause abortion, and neurological and fatal diseases in humans and in a wide range of animals. *L. monocytogenes* is a ubiquitous bacterium often isolated from different sources like food, wild animals, and the environment. Therefore, the sampling for the detection of *Lm* within the food industry, farm, and wild environment is fundamental to understand its spread and diffusion. *Lm* belongs to the genus *Listeria* together with *Listeria ivanovii* (*L. ivanovii*), pathogenic to ruminants [18], and *Listeria innocua* (*L. innocua*), considered non-pathogenic but in some cases identified as the cause of neurological disease in cattle [19].

More attention has been paid to marine ecosystems as the detection of strains of *Lm* has been reported in marine animals [20,21,22,23], which highlighted the importance of further studies of these microorganisms in relation to the environment. From a One Health perspective, it is important to consider the role of wild animals and the environment as reservoirs of bacteria and sources of infection, as the environment, animals, and humans are closely linked and interconnected [24].

Thanks to the development of genomic techniques such as the Whole Genome Sequencing (WGS), it is now possible to deeply discriminate strains to track the source of contamination and its diffusion in the environments. Indeed, WGS allows us to characterize *L. monocytogenes* and *Listeria* spp. strains evaluating sequence type (ST) and Clonal Complexes (CC) based on the similarity of the 7 housekeeping genes and clustering through analysis of the core genome multi-locus sequence typing (cgMLST) based on allelic differences. Thanks to advanced sequencing techniques it is now possible to get a deeper understanding of the genomic trait of *Listeria* spp. and *Lm*, and useful information regarding the virulence, persistence, and resistance of bacteria in relation to the environment and the host. Finally, WGS enables the monitoring of antimicrobial resistance genes to be used as a screening method to rapidly detect antimicrobial resistance [25].

This work aimed to monitor the presence of *Listeria monocytogens* and *Listeria* spp. (other than *Lm*) in sea turtle carcasses stranded along Italian coasts. The isolated strains were characterized to investigate the pathogenicity and to evaluate potential similarities between strains. Moreover, isolated strains were compared with strains from other sources such as feed, food, industrial environment samples, and clinical cases.

## 2. Materials and Methods

### 2.1. Detection of Lm and Listeria *spp.* from Sea Turtles

Between 2019 and 2022, a total of 164 carcasses of sea turtles (*C. caretta* *n* = 161 and *C. mydas* *n* = 3) stranded along Abruzzo, Molise, Campania, and Calabria coasts, were collected, and sampled for *Listeria* spp. and *L. monocytogenes* detection.

Brain and intestinal content samples were collected during necropsy examination at the Istituto Zooprofilattico Sperimentale dell’Abruzzo e Molise (IZSAM) from 89 carcasses and analyzed according to ISO11290-1:2017 [26] for detection of *Lm* and *Listeria* spp. other than *Lm*. Five suspected colonies from each potentially positive sample were selected and confirmed with MALDI-TOF MS (MALDI Biotyper^®^, Bruker Daltonics Gmbh & Co. KG, Bremen, Germany). Confirmed colonies were stored at −80 °C for Whole Genome Sequencing (WGS).

At the same time, from 2019 to 2022, the Istituto Zooprofilattico Sperimentale del Mezzogiorno (IZSM) carried out the detection of *Listeria* spp. from 75 carcasses showing fresh or moderately preserved state. From those, 75 brain tissues and 20 feces were sampled.

The detection of *Listeria* spp. was carried out with pre-enrichment of brain and fecal samples in *Listeria* Half Fraser Broth (Liofilchem s.r.l., Roseto degli Abruzzi, Italy) and subsequent enrichment in *Listeria* Fraser Broth (Liofilchem s.r.l., Roseto degli Abruzzi, Italy) at 30 °C ± 1 °C for 24 h ± 3 h. Following the streaking on *Listeria* Oxford (Liofilchem s.r.l., Roseto degli Abruzzi, Italy), two incubations at 37 °C ± 1 °C for 24 h ± 3 h and 48 h ± 3 h were performed. The typical colonies on media were isolated and streaked on non-selective culture media such as TSYEA Agar and Blood agar (Liofilchem s.r.l. Roseto degli Abruzzi, Italy), and biochemical identification tests, such as oxidase, catalase, Gram stain, haemolysis evaluation, Vitek^®^ 2 system (boiserie, Ponte a Ema, Italy) were performed. The results were confirmed by MALDI-TOF (Bruker, Germany).

One strain for each positive sample was sent, according to internal protocols, to the National Reference Laboratory for *L. monocytogenes* (NRL-Lm) of IZSAM, for WGS and further analysis.

### 2.2. Selection of Sequences Other Than Sea Turtles

Sequences for comparative analysis were selected from the bibliography. The search was done based on CC and ST similarity and sequence availability. In total, 207 assembled genome FASTA files were selected for comparative analysis, 186 belonging to a previous Italian outbreak reported by Chiaverini et al. [27], 15 strains belonging to ST204 [28], 4 belonging to ST219 [29] and 2 belonging to ST388 [30]. Details of selected sequences are reported in Supplementary data (Table S1).

### 2.3. DNA Extraction, Sequencing, and Characterization

One out of five colonies for each positive sample was chosen for DNA extraction using QIAamp^®^ DNA Mini Kit (Qiagen, Hilden, Germany) following the manufacturer’s protocol with minor modifications according to a previous study [31]. DNA quantity and quality were evaluated with a Qubit fluorometer (Thermo Fisher Scientific, Waltham, MA, USA) and Eppendorf BioSpectrometer fluorescence (Eppendorf s.r.l., Milano, Italy). The Nextera^®^ DNA Library (Illumina^®^, San Diego, CA, USA) Preparation kit was used for library preparation according to the manufacturer’s protocols, starting from 1 ng of input DNA. WGS was performed on the NextSeq^®^ 500 platform (Illumina^®^, San Diego, CA, USA) with the NextSeq 500/550 mid-output reagent cartridge v2 (300 cycles, standard 150 bp paired-end reads). For the WGS data analysis, an in-house pipeline [32] was used. The trimming step of the raw reads was performed using Trimmomatic [33] and a quality control check was performed using FastQC v.0.11.5 (https://github.com/s-andrews/FastQC (accessed on 1 September 2023)). De novo assembly of paired-end reads was carried out using SPAdes v3.11 (https://github.com/ablab/spades (accessed on 1 June 2023)) [34] with default parameters for the Illumina platform 2 × 150 chemistry. Finally, the quality check of the genome assemblies was performed with QUAST v.4.3 (https://github.com/ablab/quast (accessed on 1 September 2023)). All the genomes that met the quality parameters recommended by Timme et al. [35] were used for the subsequent analysis steps. Species were identified using Kmerfinder, available in the in-house pipeline [36,37,38].

cgMulti-locus sequence typing (cgMLST), based on the Pasteur scheme, was used to characterize *L. innocua* strains and identify the sequence type (ST) and Clonal Complex (CC) querying the Pasteur Institute platform (https://bigsdb.pasteur.fr/Listeria/ (accessed on 1 June 2023)). *L. monocytogenes* sequences were matched against the database curated by the “National Reference Centre for Whole Genome Sequencing of Microbial Pathogens: Database and Bioinformatic Analysis” (GENPAT) to evaluate the presence of matching sequences. Clustering of *Lm* strains was performed using the chewBBACA allele calling algorithm [39], available in the in-house pipeline. In agreement with the guidelines for *Lm* cgMLST typing [40], only the genomes with at least 1660 called loci (95% of the full scheme) were considered. A cut-off of 7 allele differences was used to determine the cluster [41], cgMLST tree was visualized using grapetree [42].

Genome assemblies were screened for the presence/absence of virulence, disinfectants/metal resistance, and antimicrobial resistance genes using different functions available on the BIGSdb-Lm platform (http://bigsdb.pasteur.fr/Listeria (accessed on 1 June 2023)).

Gene annotation was elaborated using PROKKA [43] and pangenome analysis using Panaroo [44]. Panaroo results were used in Scoary for the Genome-wide association study (GWAS analysis) between strains from the CC7 Italian outbreak and strain 2022.TE.25575.1.2 detected in sea turtle *C. caretta*. Panaroo results were used also to generate the Newick file using iqTree2 (version 2.2.6) [45] and tree visualized using iTol v6 [46].

Plasmids check was performed using PlasmidFinder 2.1 (accessed online 1 September 2023) [47,48] with default parameters.

Gene analysis and gene annotation visualization were elaborated using Rstudio v. 4.3.1 [49] and visualized with iTol v6.

## 3. Results

### 3.1. Strain Selection and Identification

Colonies suspected to belong to *Listeria* spp. were detected phenotypically in samples collected from 7 specimens: 6 from *C. caretta* and 1 from *C. mydas*. In particular, 3 strains of *L. monocytogenes*, 2 of *L. innocua*, and 1 of *L. ivanovii* were detected in *C. caretta*; while 1 strain of *L. monocytogenes* and 1 of *L. innocua* were isolated from *C. mydas* (Table 1). Interestingly, both *L. monocytogenes* and *L. innocua* were isolated from one individual of *C. mydas* (ID 294624).

The necropsy reported for ID 49074 and ID 109137 showed splenomegaly, granulomatosis in the spleen and sero-hemorragic pericardial effusion; moreover, meningeal congestion was also observed in the individual ID 109137.

Septicemic lesions in ID 288887 were reported by Di Renzo et al. [21]. Lesions reported at postmortem inspection were not related to listeriosis for ID 316173, 316953, and 53724. Moreover, specimen ID 294624 showed a high level of cadaveric decomposition and did not allow to link lesions to the presence of *L. monocytogenes* and *L. innocua*.

### 3.2. Listeria monocytogenes and Other Listeria *spp.* Characterization and Pangenome Analysis

*L. monocytogenes* strain genomes ranged from 2,853,152 bp to 2,931,008 bp; the *L. innocua* from 2,856,977 bp to 2,839,444 bp; and *L. ivanovii* was 2,892,978 bp. The GC content was highest in *L. monocytogenes* (37.7–37.9%), compared to *L. innocua* and *L. ivanovii* (respectively 37.0–37.3%).

Multilocus sequence typing (MLST) analysis characterized *L. monocytogenes* strains as ST7 (CC7) for the strain ID 2022.TE.25575.1.2, ST388 (CC388) for 2022.TE.32578.1.2, ST204 (CC204) for 2022.TE.37095.1.2, and ST219 (CC4) for 2023.TE.2348.1.2. While *L. innocua* was identified as ST536 (CC133) for 2022.TE.37093.1.2, ST542 (CC542) for 2022.TE.37041.1.2, and ST1481 (CC1481) for 2022.TE.37094.1.2. (Table 1).

The total pangenome analysis between the 8 strains (*L. monocytogenes* and other *Listeria* spp.) revealed 4035 gene clusters sequences (CDS): 2033 were core genes in all strains, 1004 shell genes, and 998 cloud strains. No soft-core strains were detected.

*L. monocytogenes* shared a total of 425 genes with *L. innocua*, and only 36 with *L. ivanovii*. This is supported by the likelihood of these species being closer evolutionary [50] (Figure S1).

**Figure 1:**
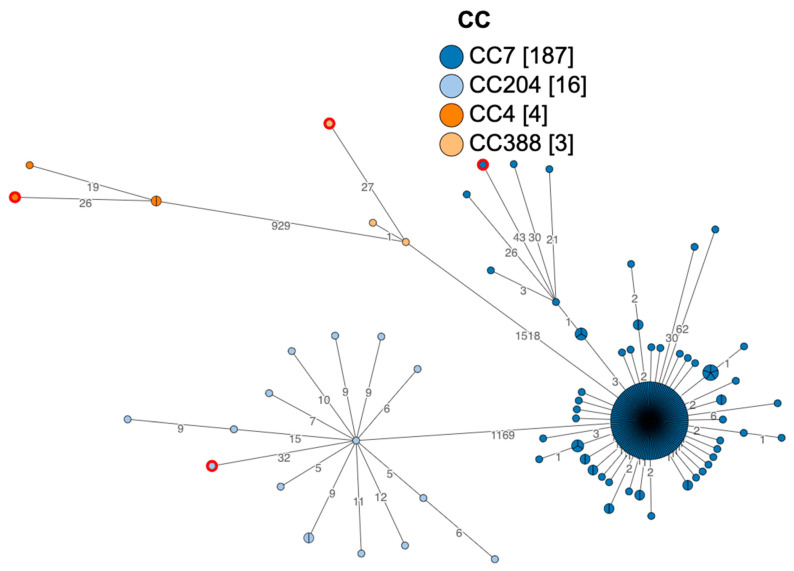
cgMLST tree. In blue strains belonging to CC7, the CC7 strain isolated from marine turtle are displayed with a red circular line. In light blue and red line strain isolated from marine turtle CC204, in orange and red line strain isolated from marine turtle CC388, in yellow, and with a red circular line, strain isolated from marine turtle CC4.

The cgMLST analysis of selected sequences between strains isolated from sea turtles and human clinical cases, food, or environment showed an allelic difference greater than 7 alleles hence no clustering was detected (Figure 1).

### 3.3. Gene Distribution

#### 3.3.1. Virulence Genes

**Figure 2:**
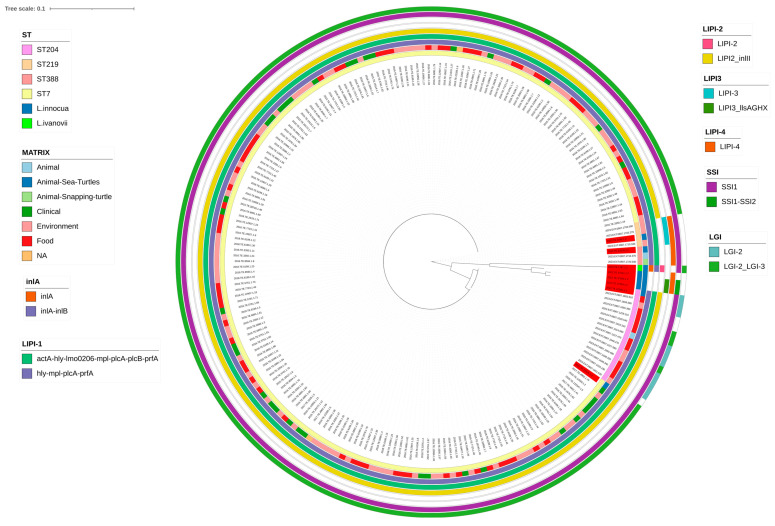
Circular phylogenetic tree visualisation and relative information. The phylogenetic tree represents all strains used in the present study. In red are marked strains collected from sea turtle. The relative information for each strain are displayed from the inside to the outside layer as following: ST, Source of isolates, virulence gene inlA and inlB, LIPI-1 islet (in green virulence gene actA, hly, lmo0206, mpl, plcA, plcB and prfA. In purple hly, mpl plcA and prfA). LIPI-3 (in light blue strains where all genes of LIPI-3 were present, in green strains where only LIPI3_llsA, LIPI3_llsG, LIPI3_llsH, LIPI3_llsX were present), LIPI-2 (in pink: strain where all genes from LIPI-2 were present in yellow strains were only LIPI2_inlll was present), LIPI-4, SSI (In purple strains were all genes forming SSI1 islet were present, in green where both SSI and SSI2 were present), LGI (in light blue strains where all genes of LGI-2 were present in green where both were present). In white are represented missing genes.

Virulence genes analysis allowed the identification of shared genes among the strains belonging to the species of sea turtles (Figure 2). In total, 35 virulence genes were shared among all the strains, 54 virulence genes were shared within the *L. monocytogenes* and 39 virulence genes within the *L. innocua* species. Among the major virulence genes *hly* as well as *inlA* and *InlB* were present in *L. monocytogenes* only *inlA* in *L. ivanovii*, neither of them in *L. innocua*. No premature stop codes were found in inlA of *L. monocyogenes*. Most of the internalin genes were present in *L. monocytogenes*, but not in the other *Listeria* spp. investigated. The gene *lap* was present in all the examined *Listeria* spp. but *lapB* was present only in *L. monocytogenes* strains.

All four strains of *L. monocytogenes* shared LIPI-1 (*actA*, *hly*, *mpl, plcA, plcB, prfA* and *lmo0206* or *orfX*) virulence genes, but only *mpl*, *hly*, *prfA*, and *plcA*, were present in *L. ivanovii*, none of them were present in *L. innocua*.

All genes from LIPI-2 were present in *L. ivanovii* strain, but not in the other species except for LIPI-2_*inlll* present in two *L. monocytogenes* isolated from sea turtles (ST7 and ST204). LIPI-3 was present in only one *L. monocytogenes* ST219, and some genes belonging to LIPI-3 were present in all strains of *L. innocua*, particularly, *llsA, llsG, llsH* and *llsX. LisK* and *lisR* were present in all the strains investigated. The genes LIPI-4 (group LM9005581) were present only in 2 strains (2022.TE.32578.1.2 and 2022.TE.2348.1.2) out of 4 of *L. monocytogenes* and in all the *L. innocua* strains (Figure 2).

All isolated strains carried genes for arsenic and cadmium resistance *LGI-2_LMSO2310*. *L. ivanovii* carried also the *LGI-2_LMOSA2320* gene and *LGI-3* genes (*LGI-3_LmUB3PA_1705*, *LGI-3_LmUB3PA_1706*). Only six *L. monocytogenes* ST204 strains isolated from food, environment, and animals, including the strain isolated from the sea turtle carried all LGI-2 genes. *L. monocytogenes* ST388 and ST219 carried only *LGI-2_LMSO2310*. *L. monocytogenes* ST7 compared to those isolated from other ST, carried less LGI-2 genes and more genes belonging to *LGI-3*. Noteworthy, ST7 isolated from clinical cases and sea turtles were sharing the same pattern, carrying only *LGI-2_LMSO2310* and *LGI-3_LmUB3PA_16850*.

**Figure 3:**
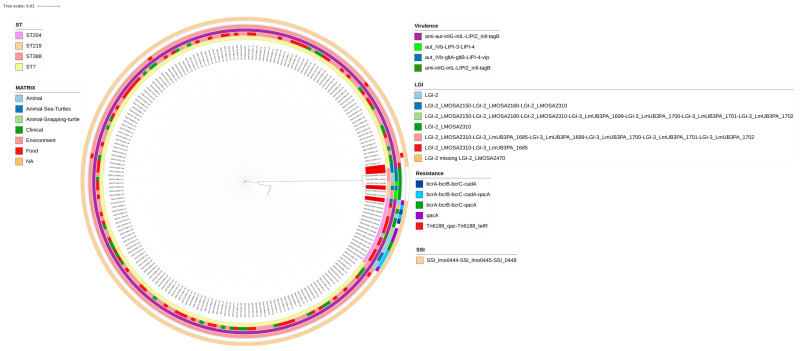
Gene presence visualization. In red isolates from sea turtles. In order from the outer layer to inside: sequence type, matrix of origin of the strain sequences, virulence, resistance, stress survival island genes. On the first layer in bright pink there are isolates ST204, in dark yellow ST219, in pink ST388 and in yellow ST7. On the second layer, light blue represents isolates detected in animals, in blue sea turtles, in light green Snipping turtle, in green clinical isolates, in pink environment, in red food, in dark yellow source unknown. The third layers represent virulence genes. In purple thereare isolates carrying ami, aut, inlG, inlL, LIPI2_inlll and tagB genes. In blue there are isolates carrying aut_Ivb, gtlA, gtlB, LIPI-4 and vip genes, in light green there are gene aut_Ivb, LIPI-3 and LIPI-4 and dark green aut, inlG, inlL, LIPI2_inll and tagB genes. On the fourth layer there is LGI, in light blue there are all strains carrying LGI-2 genes, in dark blue we found isolates carrying only part of the LGI-2 genes (LGI-2_LMOSA2150, LMOSA2310, LMOSA2160). In light green we found LGI-2 genes and LGI-3. In green we found isolates carrying only LGI-2_LMSO2310. In pink there are genes LGI-3 and only Lgi-2_LMSOA2310. In red there are genes isolates carrying only LGI-2_LMSO2310 and LGI-3_LmUB3PA_1685. In yellow, we found isolates carry all genes LGI-2 except LGI_LMOSA2470. Fifth layer Resistance genes in blue bcrA, bcrB, bcrC and cadA, in light blue bcrA, bcrB, bcrC, cadA and qacA, in green bcrA, bcrB, bcrC and qacA, in purple qacA and in red TN6188_qac and Tn6188_TetR. Outter layer SSI genes in light yellow. In white missing genes.

The distribution of virulence genes within *L. monocytogenes* showed that ST204 and ST219 shared genes *autIVb* and LIPI-4. Instead, LIPI-2_*inlll, tagB, inlL* and *inlG* were common to all CC7, only one CC7 strain did not carry the gene *ami*, present in the majority of the other CC7 strains instead, and no differences were reported between the marine strains and other sources. LIPI-3, associated with increased virulence in *L. monocytogenes*, was present in sequences belonging to ST219, but not from the sea turtle. The strain isolated from sea turtles showed *gltA* and *gltB* genes, which were absent in the other strain within ST219 isolated from the environment (Figure 3).

#### 3.3.2. Antimicrobial Resistance (AMR) Genes

No major differences were reported within AMR genes. Indeed, all isolated strains of *L. monocytogenes* and other *Listeria* spp. shared genes *fosX* (resistance to fosfomycin), *lmo0919* (resistance to lincosamides), *norB* (resistance to quinolones), *sul* (resistance to sulfonamides), and *lmo1695* (resistance to cationic antimicrobials peptides, CAMPs).

#### 3.3.3. Listeria Genomic Island (LGI)

All isolated strains carried genes for arsenic and cadmium resistance *LGI-2_LMSO2310*. *L. ivanovii* carried also *LGI-2_LMOSA2320* gene and *LGI-3* (LGI-3_LmUB3PA_1705, LGI-3_LmUB3PA_1706) genes. Only 6 *L. monocytogenes* ST204 strains isolated from food, environment, and animals including the strain isolated from the sea turtle carried all genes LGI-2.

*L. monocytogenes* ST7 strains compared to those isolated from other ST, carried less LGI-2 genes and more genes belonging to *LGI-3*. Noteworthy, ST7 strains isolated from clinical cases and sea turtles shared the same pattern, carrying only LGI-2_LMSO2310 and LGI-3_LmUB3PA_16850.

#### 3.3.4. Disinfectant Resistance Genes

Among the isolated strains, only *L. monocytogenes* ST204 carried gene *qacA* involved in disinfectant resistance to benzalkonium chloride (BC), this gene was present also in other strains ST204 isolated from food, environment, and animal samples.

#### 3.3.5. Stress Survival Islets

All strains shared genes *lmo1799* and *lmo1800*, but only two *L. innocua* presented genes *SSI2_lmo0464* and *SSI2_lmo0465*. All the strains except one *L. innocua* presented the gene *SSI1_lmo0447* as well as the sequence compared to other sources. Genes *SSI1_lmo0444*, *SSI1_lmo0445*, *SSI1_lmo0446* and *SSI1_lmo0448* were found only in two out of four strains of *L. monocytogenes*, ST204 and ST7 (Figure 2). Noteworthy, this pattern was similar to the strains collected from other sources of the same ST.

#### 3.3.6. GWAS Analysis

GWAS analysis did not highlight any relevant unique gene that may explain differences between terrestrial and marine isolated strains, although it highlighted a total of 60 genes missing in the CC7 strain isolated from *C. caretta* and in 2–4% of the strains from the clinical outbreak in Italy. Mainly they were genes annotated as hypothetical proteins, cadmium resistance proteins, ATP-dependent protease, family transposase, NADH peroxidase, multicopper oxidase (MCO), and DNA-invertase hin and elongation factor Ts mitochondrial (Table S3).

## 4. Discussion

The presence of *Listeria* spp. strains (including *Lm*) in sea turtles stranded along Italian coasts was not always linkable to lesions ascribable to listeriosis. The analysis of those isolates demonstrated that among the 164 animals examined, the prevalence of *L. monocytogenes* was about 1.8%. However, this was calculated considering that, between the detected strains, *L. innocua* is considered less pathogenic than *L. monocytogenes* and *L. ivanovii*. Moreover, one strain of *L. monocytogenes* was detected in a sample with advanced post-mortem alteration. Therefore, only 3 *L. monocytogenes* and 1 *L. ivanovii* were accounted as possible causes of the disease in the sea turtles examined.

*L. innocua* is closely related to *L. monocytogenes* [51]; as showed through the pangenome analysis, 425 genes were shared between *L. monocytogenes* and *L. innocua,* while only 36 genes were shared between *L. monocytogenes* and *L. ivanovii.*

Furthermore, *L. ivanovii* shares virulence genes with *L. monocytogenes*, as well as atypical *L. innocua*, which shows potential virulence [52,53,54].

The application of molecular techniques, particularly WGS, allows deep discrimination between isolates, and it is fundamental for clustering and epidemiological investigation [24]. Moreover, WGS is useful to get a deeper understanding of the gene distribution between different sources. In our study, we characterized *L. monocytogenes* ST7, ST204, ST219, and ST388, respectively CC7, CC204, CC4, and CC388. Hence, no clustering was detected among the isolates. The ST detected in our study are commonly found from other sources, indeed ST204 is reported to have been isolated generally from food sources [55], while the other STs have been previously reported in human outbreaks. In particular, ST219, or CC4, is widely reported as a hypervirulent clone of *L. monocytogenes* associated with human outbreaks of listeriosis [56]. Strain ST388, which was isolated from a case of listeriosis in a loggerhead sea turtle in Italy [21], was reported also in Spain as the cause of disease in humans [57]. *L. monocytogenes* ST7 was reported as the cause of an outbreak in Italy between 2015 and 2016 [27]. Further analysis between the strain isolated from the sea turtle and the strains causing the outbreak did not show any correlation, since allelic differences were greater than 7, hence no cluster was identified.

*L. monocytogenes*, *L. innocua* and, *L. ivanovii* shared virulence genes such as those for adhesion and invasion (*fbpAt, lap, lpeA, lspA*), those for teichoic acid glycosylation of bacteria walls (*gtcA*), *oatA* for resistance to different antimicrobial compounds and also required for the growth inside macrophages, *PdgA* for resistance to lysozyme [18], and *prsA2*, which is involved in the correct folding of proteins, hence maturation and secretion of some virulence proteins, and important for cell viability [58].

Regarding the differences in virulence gene distribution, *L. monocytogenes* isolates shared *inlA* with *L. ivanovii* [58], not present in *L. innocua* instead. Gene cluster LIPI-1 was present in all *L. monocytogenes*, but only *hly*, *plcA, prfA* and *mpl* genes were present in *L. ivanovii*. The presence of these genes was associated with atypical strains of *L. innocua* as well [54], indeed, in our case, the gene cluster LIPI-1 was absent in *L. innocua*. LIPI-2, as expected, was found only in *L. ivanovii*, being this gene cluster-specific for this species.

In our study, only one *L. monocytogenes* strain (CC4 ST219) and two of *L. innocua* presented the genes LIPI-3, while LIPI-4 genes were present in *L. monocytogenes* ST388, ST219 (CC4), and all the *L. innocua*. LIPI-3 contains genes encoding listeriolysinS (LLS) (LIPI-3_*llsA*) and is reported also to play a role in gut colonization in *L. monocytogenes.* LIPI-3, together with genes belonging to LIPI-4 (usually associated with *L. monocytogenes* strains with high neuronal and placental tropism) are indicators of hypervirulent *L. monocytogenes* strains [41]. Interestingly, LIPI-3 and LIPI-4 are commonly reported in *L. innocua* [52,53,54,55,56,57]. Indeed LIPI-4 is important in the carbon metabolism in saprophytic environments [59]. Antimicrobial resistance genes confirmed what was reported in previous studies [59]. Noticeably, no resistance to penicillin was reported, confirming penicillin and gentamycin as first-line antimicrobials to be used in case of listeriosis [60].

The resistance gene for arsenic and cadmium *LGI-2_LMSO2310* was present in all strains investigated. Stress survival islets (SSIs) encode genetic mechanisms for stress resistance to temperature, pH, and osmotic stress. *Lmo1799* and *Lmo1800* were found in all the strains, while SSI-1 genes were more common in *L. monocytogenes* than SSI-2, which were found only in 2 strains of *L. innocua*. This confirmed what was reported by Palaiodimou et al. [53].

The presence of *qacA* gene for resistance to benzalkonium chloride (BC) and cadmium [61,62] was reported only in the ST204 strain. BC is a disinfectant compound usually used for sanitizing procedures in food environments. The presence of *qacA* gene in the ST204 strain could not be the case as ST204 is usually associated with food environment and food [55].

No major differences were noted among marine turtle strains and those isolated from other sources such as animals, clinical cases, food, and environments. GWAS analysis as well, could not find unique genes in the genome between strains from terrestrial and marine environments. Virulence, LGI, and SSI gene distribution patterns for strain ST7 (2022.TE.25575.1.2) isolated from the sea turtle showed no difference from strains isolated from an Italian outbreak in the Marche region between 2015 and 2016 [27]. However, the ST7 strain (2022.TE.25575.1.2) was isolated from a carcass collected in Falerna (CZ), showing siero hemorrhagic pericardial fluid, meningeal congestion and splenomegaly. Moreover, the cgMLST analysis showed an allelic distance higher than 7 alleles, which does not allow to cluster this strain with the one from the outbreak.

*L. monocytogenes* is an important public health issue, and from a “One Health Approach” perspective, WGS applied for genome characterization of *L. monocytogenes* strains can support the study of the distribution of virulence and resistance genes, associated with the ability of *L. monocytogenes* to develop disease between different sources. For instance, the presence of LIPI-4 is usually associated with hypervirulent strains, even more CC4 are usually detected in clinical cases. In our case, strain CC4 (2023.TE.2348.1.2) could be suspected to be potentially hypervirulent, considering the presence of multiple virulence genes. Nevertheless, only generalized macroscopic lesions were seen at post-mortem such as enlarged spleen, splenic granulomatosis, and siero hemorrhagic pericardial fluid. Therefore, it was impossible to confirm listeriosis as the cause of death also because no lesions were reported in the brain. This might indicate that a combination of virulence factors is necessary for the development of the disease, among them the health status of the animal is a critical factor. Still, the examination of the brain and its histological exams are of relevant importance when investigating a suspected case of listeriosis.

*L. ivanovii* strain, on the other hand, was isolated from the brain, although no lesions were found. Still, it would be interesting to understand the role of *L. ivanovii* in sea turtles, and if it can be the cause of disease like in ruminants.

Despite the above-mentioned results, a limit of this study was the restricted number of strains that we could collect (8 strains in total). To get a clearer and more comprehensive picture of this matter, a bigger dataset is needed.

## 5. Conclusions

It is known that *L. monocytogenes*, *L. innocua*, and *L. ivanovii* can adapt to the marine environment, and that *L. monocytogenes* can be another cause of disease in marine animals as reported previously by Di Renzo [21]. This work represents the results of three years of monitoring of *Lm* and *Listeria* spp. along the Italian sea coast.

In our study, we highlighted the presence of strains of *L. monocytogenes* in the marine environment, and we compared them to those isolated from other sources, investigating the distribution of the genes involved in abortion and neurological diseases usually associated with human outbreaks. Furthermore, we pointed out the presence of *L. innocua* and *L. ivanovii* in the marine environment, the latter known for its potential to cause disease in ruminants.

The application of WGS allowed us to characterize the detected strains and highlight similarities. No clusters were reported, but we confirmed the presence of heterogeneity strains of *L. monocytogenes* in the marine environment as well as in the terrestrial one. Furthermore, the detection of CC in the worldwide marine environment, associated with clinical cases is itself an interesting finding, which leads to consider the role of anthropogenic activities in the marine environment. In support of this thesis, we found similarities in the distribution of virulence and resistance genes between our strains of *L. monocytogenes* and those taken from the literature, strains usually linked to clinical cases and persistent strains detected in the food industry.

We also aimed to investigate potential differences between strains found in the marine environment compared to the terrestrial one. We found a strain of *L. monocytogenes CC7* with a genomic pattern like those reported in an Italian outbreak, but the GWAS analysis did not allow us to determine unique genes that supported our goal.

In conclusion, the genomic analysis did not show major differences between terrestrial and marine strains. However, from a One Health perspective, our study highlighted the importance of investigating *L. monocytogenes* in wildlife to understand its distribution including in the marine environment.

In conclusion, those animals could have been sick and, at the same time carrying the pathogen. Furthermore, although the immune system of sea turtles is still not completely understood, it is known that the presence of environmental contaminants can play a crucial role in the health status of the animals. The evaluation of more important contaminants could be interesting to deeply understand their role in the development of the disease.

In a forward-looking perspective, it would be interesting to collect more data to evaluate the marine environment’s impact on human activity and viceversa, investigate the pathogenicity in aquatic animals of other *Listeria* species other than *L. monocytogenes* such as *L. ivanovii*, and explore the role of marine animals as carriers and reservoirs of the pathogen in the environment. Finally, it is important to acknowledge the presence of genes shared between marine and terrestrial environments to search for common paths that may explain their distribution.

## Figures and Tables

**Table 1:** Summary of *Liseteria* spp. isolated from sea turtles in Italy in 2021 and 2022.

Laboratory	Animal ID	Strains_ID	Year	Location	Species	Sex	Maldi-TOF	Matrix	Serotype	ST (CC)
IZSAM	288887	2022.TE.32578.1.2	2021	Ortona (CH)	*C. caretta*	Female	*L. monocytogenes*	multiorgans	4b	ST388 (CC388)
	316953	2022.TE.37041.1.2	2021	Pescara (PE)	*C. caretta*	Female	*L. innocua*	feces	NA	ST542 (CC542)
	316173	2022.TE.37093.1.2	2021	Pescara (PE)	*C. caretta*	Female	*L. innocua*	brain	NA	ST536 (CC133)
	294624	2022.TE.37094.1.2	2021	Montesilvano (PE)	*C. mydas*	Female	*L. innocua*	feces	NA	ST1481 (CC1481)
	294624	2022.TE.37095.1.2	2021	Montesilvano (PE)	*C. mydas*	Female	*L. monocytogenes*	feces	1/2a	ST204 (CC204)
	53724	2023.TE.1767.1.2	2022	Montesilvano (PE)	*C. caretta*	Female	*L. ivanovii*	brain	NA	NA
IZSM	109137	2022.TE.25575.1.2	2021	Falerna (CZ)	*C. caretta*	Female	*L. monocytogenes*	brain	1/2a	ST7 (CC7)
	49074	2023.TE.2348.1.2	2022	Anacapri (NA)	*C. caretta*	Male	*L. monocytogenes*	brain/faeces	4b	ST219 (CC4)

## Data Availability

At the moment of submission, the authors will submit the genomic sequencing to NCBI as soon as possible.

## Supplementary Materials

The following supporting information can be downloaded at: https://www.mdpi.com/article/10.3390/microorganisms12040817/s1, Figure S1: Pangenome analysis of the isolates in this study, grouped by species. Numbers represent gene coding loci associated with one or more species; Table S1: Sequences used in the study. (FPP-Food Processing Environment; RS-Retail Store; PH- Patient’s House; NA-Not Applicable); Table S2: Gene presence between sea turtle strains; Table S3: GWAS CC7.
