# Supplementary material for: Genomic Characterization of Listeria monocytogenes and Other Listeria Species Isolated from Sea Turtles
Source: Microorganisms. 2024 Apr 18;12(4):817. doi: 10.3390/microorganisms12040817 (PMC11052188; doi:10.3390/microorganisms12040817)
Supplement: Supplementary file 1 [file microorganisms-12-00817-s001.zip › microrganism 2912735_Figure S1. Pangenome analysis of the isolates in this study, grouped by species..pdf]

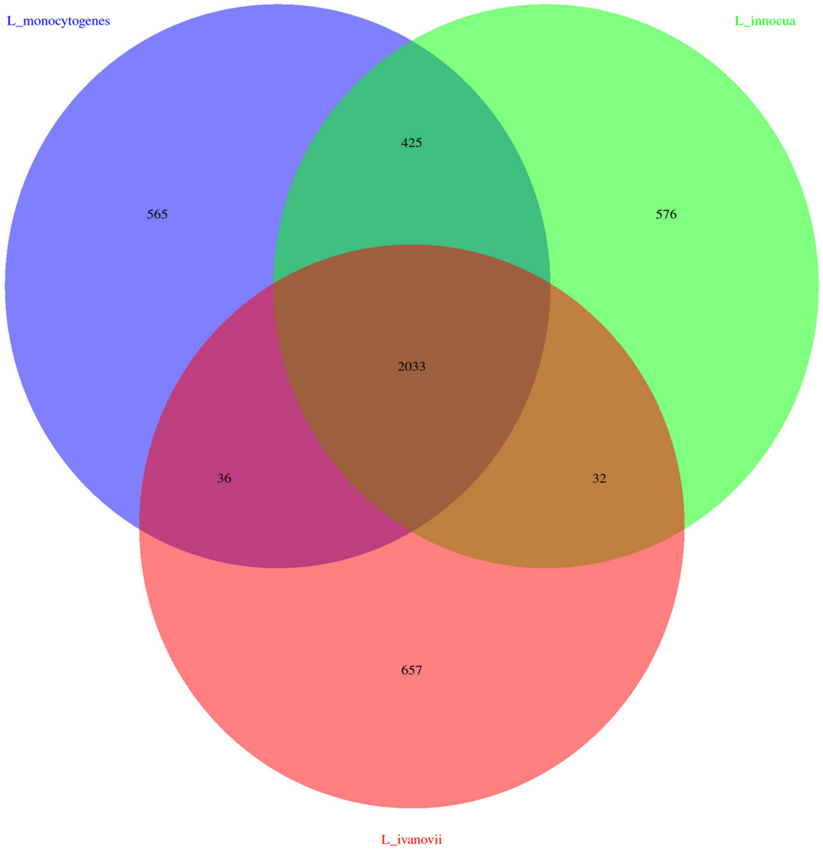

Figure S1. Pangenome analysis of the isolates in this study, grouped by species. Numbers represent gene coding loci associated with one or more species
