# Supplementary material for: Genomic Characterization of Listeria monocytogenes and Other Listeria Species Isolated from Sea Turtles
Source: Microorganisms. 2024 Apr 18;12(4):817. doi: 10.3390/microorganisms12040817 (PMC11052188; doi:10.3390/microorganisms12040817)
Supplement: Supplementary file 1 [file microorganisms-12-00817-s001.zip › microrganism 2912735_Table S1. Sequences used in the study..pdf]

Table S1. Sequences used in the study. (FPP-Food Processing Environment; RS-Retail Store; PH- Patient's House; NA-Not Applicable).

ID	Matrix	Year	ID Source.	Bioproject	Biosample	Reference	ST
2016.TE.12559.1.2	Environment	2016	NA	/	/	/	7
2017.TE.4902.1.70	Clinical	/	NA	/	/	/	7
2020.TE.122547.1.5	Clinical	2020	NA	/	/	/	7
2017.TE.4902.1.41	Clinical	/	NA	/	/	/	7
2017.TE.4902.1.56	Clinical	/	NA	/	/	/	7
2016.TE.12907.1.27	Salami	2016	FPP1	PRJNA750067	SAMN20429606	Chiaverini et al., 2021	7
2016.TE.12907.1.26	Salami	2016	FPP1	PRJNA750067	SAMN20429607	Chiaverini et al., 2021	7
2018.TE.6199.1.25	Environment	2018	RS5	PRJNA750067	SAMN20429608	Chiaverini et al., 2021	7
2018.TE.6199.1.23	Environment	2018	RS5	PRJNA750067	SAMN20429609	Chiaverini et al., 2021	7
2018.TE.6199.1.24	Environment	2018	RS5	PRJNA750067	SAMN20429610	Chiaverini et al., 2021	7
2018.TE.6199.1.26	Environment	2018	RS5	PRJNA750067	SAMN20429611	Chiaverini et al., 2021	7
2018.TE.6199.1.29	Environment	2018	RS5	PRJNA750067	SAMN20429612	Chiaverini et al., 2021	7
2018.TE.6199.1.32	Environment	2018	RS5	PRJNA750067	SAMN20429613	Chiaverini et al., 2021	7
2016.TE.6891.1.69	Salami	2016	FPP2	PRJNA750067	SAMN20429614	Chiaverini et al., 2021	7
2016.TE.6891.1.71	Salami	2016	FPP2	PRJNA750067	SAMN20429615	Chiaverini et al., 2021	7
2016.TE.7723.1.40	Hog head cheese	2016	FPP1	PRJNA750067	SAMN20429616	Chiaverini et al., 2021	7
2016.TE.3350.1.35	Environment	2016	FPP1	PRJNA750067	SAMN20429617	Chiaverini et al., 2021	7
2016.TE.1976.1.83	Clinical	2015	NA	PRJNA750067	SAMN20429618	Duranti et al., 2018	7
2015.TE.28740.1.45	Clinical	2015	NA	PRJNA750067	SAMN20429619	Duranti et al., 2018	7
2016.TE.8594.1.4	Environment	2016	FPP3	PRJNA750067	SAMN20429620	Chiaverini et al., 2021	7
2018.TE.5686.1.4	Environment	2018	PH2	PRJNA750067	SAMN20429621	Chiaverini et al., 2021	7
2018.TE.6199.1.12	Environment	2018	PH2	PRJNA750067	SAMN20429622	Chiaverini et al., 2021	7
2018.TE.6199.1.13	Environment	2018	PH2	PRJNA750067	SAMN20429623	Chiaverini et al., 2021	7
2018.TE.6199.1.30	Environment	2018	RS5	PRJNA750067	SAMN20429624	Chiaverini et al., 2021	7
2016.TE.2578.1.69	Clinical	2016	NA	PRJNA750067	SAMN20429625	Chiaverini et al., 2021	7
2016.TE.3350.1.48	Environment	2016	FPP1	PRJNA750067	SAMN20429626	Chiaverini et al., 2021	7
2016.TE.3350.1.49	Environment	2016	FPP1	PRJNA750067	SAMN20429627	Chiaverini et al., 2021	7
2016.TE.3350.1.36	Environment	2016	FPP1	PRJNA750067	SAMN20429628	Chiaverini et al., 2021	7
2016.TE.3350.1.37	Environment	2016	FPP1	PRJNA750067	SAMN20429629	Chiaverini et al., 2021	7

2016.TE.5761.1.61	Clinical	2016	NA	PRJNA750067	SAMN20429630	Duranti et al., 2018	7
2016.TE.10956.1.20	Environment	2016	FPP3	PRJNA750067	SAMN21220761	Chiaverini et al., 2021	7
2016.TE.3350.1.45	Environment	2016	FPP1	PRJNA750067	SAMN20429631	Chiaverini et al., 2021	7
2016.TE.7723.1.45	Hog head cheese	2016	FPP1	PRJNA750067	SAMN20429632	Chiaverini et al., 2021	7
2016.TE.3350.1.39	Environment	2016	FPP1	PRJNA750067	SAMN20429633	Chiaverini et al., 2021	7
2016.TE.10956.1.26	Environment	2016	RS2	PRJNA750067	SAMN21220762	Chiaverini et al., 2021	7
2015.TE.19114.1.9	Clinical	2015	NA	PRJNA750067	SAMN20429634	Chiaverini et al., 2021	7
2016.TE.7723.1.55	Hog head cheese	2016	FPP1	PRJNA750067	SAMN20429635	Chiaverini et al., 2021	7
2016.TE.7723.1.49	Hog head cheese	2016	FPP1	PRJNA750067	SAMN20429636	Chiaverini et al., 2021	7
2016.TE.1976.1.84	Clinical	2015	NA	PRJNA750067	SAMN20429637	Duranti et al., 2018	7
2016.TE.1976.1.85	Clinical	2015	NA	PRJNA750067	SAMN20429638	Chiaverini et al., 2021	7
2015.TE.18427.1.10	Clinical	2015	NA	PRJNA750067	SAMN20429639	Chiaverini et al., 2021	7
2015.TE.18427.1.4	Clinical	2015	NA	PRJNA750067	SAMN20429640	Chiaverini et al., 2021	7
2015.TE.18427.1.6	Clinical	2015	NA	PRJNA750067	SAMN20429641	Duranti et al., 2018	7
2015.TE.18427.1.8	Clinical	2015	NA	PRJNA750067	SAMN20429642	Duranti et al., 2018	7
2015.TE.19114.1.7	Clinical	2015	NA	PRJNA750067	SAMN20429643	Duranti et al., 2018	7
2015.TE.28740.1.46	Clinical	2015	NA	PRJNA750067	SAMN20429644	Chiaverini et al., 2021	7
2015.TE.29172.1.7	Clinical	2015	NA	PRJNA750067	SAMN20429645	Duranti et al., 2018; Orsini et al., 2018a (CP014790)	7
2015.TE.31940.1.12	Clinical	2015	NA	PRJNA750067	SAMN20429646	Duranti et al., 2018	7
2016.TE.10956.1.21	Environment	2016	RS2	PRJNA750067	SAMN20429647	Chiaverini et al., 2021	7
2016.TE.10956.1.22	Environment	2016	RS2	PRJNA750067	SAMN20429648	Chiaverini et al., 2021	7
2016.TE.10956.1.23	Environment	2016	RS2	PRJNA750067	SAMN20429649	Chiaverini et al., 2021	7
2016.TE.10956.1.24	Environment	2016	RS2	PRJNA750067	SAMN20429650	Chiaverini et al., 2021	7
2016.TE.10956.1.27	Environment	2016	RS2	PRJNA750067	SAMN20429651	Chiaverini et al., 2021	7
2016.TE.10956.1.28	Environment	2016	RS2	PRJNA750067	SAMN20429652	Chiaverini et al., 2021	7
2016.TE.10956.1.29	Environment	2016	RS2	PRJNA750067	SAMN20429653	Chiaverini et al., 2021	7
2016.TE.10956.1.30	Environment	2016	RS2	PRJNA750067	SAMN20429654	Chiaverini et al., 2021	7
2016.TE.10956.1.5	Bresaola	2016	PH1	PRJNA750067	SAMN20429655	Chiaverini et al., 2021	7
2016.TE.10956.1.6	Bresaola	2016	PH1	PRJNA750067	SAMN20429656	Chiaverini et al., 2021	7
2016.TE.10956.1.7	Bresaola	2016	PH1	PRJNA750067	SAMN20429657	Chiaverini et al., 2021	7

2016.TE.12907.1.13	Pancetta	2016	FPP1	PRJNA750067	SAMN20429658	Chiaverini et al., 2021	7
2016.TE.12907.1.14	Pancetta	2016	FPP1	PRJNA750067	SAMN20429659	Chiaverini et al., 2021	7
2016.TE.12907.1.16	Pancetta	2016	FPP1	PRJNA750067	SAMN20429660	Chiaverini et al., 2021	7
2016.TE.12907.1.19	Pancetta	2016	FPP1	PRJNA750067	SAMN20429661	Chiaverini et al., 2021	7
2016.TE.12907.1.23	Salami	2016	FPP1	PRJNA750067	SAMN20429662	Chiaverini et al., 2021	7
2016.TE.12907.1.24	Salami	2016	FPP1	PRJNA750067	SAMN20429663	Chiaverini et al., 2021	7
2016.TE.12907.1.25	Salami	2016	FPP1	PRJNA750067	SAMN20429664	Chiaverini et al., 2021	7
2016.TE.12907.1.28	Salami	2016	FPP1	PRJNA750067	SAMN20429665	Chiaverini et al., 2021	7
2016.TE.12907.1.29	Salami	2016	FPP1	PRJNA750067	SAMN20429666	Chiaverini et al., 2021	7
2016.TE.12907.1.30	Salami	2016	FPP1	PRJNA750067	SAMN20429667	Chiaverini et al., 2021	7
2016.TE.12907.1.31	Salami	2016	FPP1	PRJNA750067	SAMN20429668	Chiaverini et al., 2021	7
2016.TE.15639.1.22	Environment	2016	RS2	PRJNA750067	SAMN20429669	Chiaverini et al., 2021	7
2016.TE.15639.1.26	Environment	2016	RS2	PRJNA750067	SAMN21220763	Chiaverini et al., 2021	7
2016.TE.15827.1.11	Pancetta	2016	FPP1	PRJNA750067	SAMN20429670	Chiaverini et al., 2021	7
2016.TE.15827.1.15	Loin	2016	FPP1	PRJNA750067	SAMN20429671	Chiaverini et al., 2021	7
2016.TE.15827.1.6	Loin	2016	FPP1	PRJNA750067	SAMN20429672	Chiaverini et al., 2021	7
2016.TE.1976.1.81	Clinical	2015	NA	PRJNA750067	SAMN20429673	Duranti et al., 2018	7
2016.TE.1976.1.82	Clinical	2015	NA	PRJNA750067	SAMN20429674	Duranti et al., 2018	7
2016.TE.1976.1.90	Clinical	2016	NA	PRJNA750067	SAMN20429675	Duranti et al., 2018	7
2016.TE.2578.1.71	Clinical	2016	NA	PRJNA750067	SAMN20429676	Duranti et al., 2018	7
2016.TE.2578.1.76	Hog head cheese	2016	RS1	PRJNA750067	SAMN20429677	Chiaverini et al., 2021	7
2016.TE.3350.1.10	Clinical	2016	NA	PRJNA750067	SAMN20429678	Duranti et al., 2018	7
2016.TE.3350.1.11	Environment	2016	FPP1	PRJNA750067	SAMN20429679	Chiaverini et al., 2021	7
2016.TE.3350.1.12	Environment	2016	FPP1	PRJNA750067	SAMN20429680	Chiaverini et al., 2021	7
2016.TE.3350.1.18	Environment	2016	FPP1	PRJNA750067	SAMN20429681	Chiaverini et al., 2021	7
2016.TE.3350.1.19	Environment	2016	FPP1	PRJNA750067	SAMN20429682	Chiaverini et al., 2021	7
2016.TE.3350.1.24	Environment	2016	FPP1	PRJNA750067	SAMN20429683	Chiaverini et al., 2021	7
2016.TE.3350.1.27	Environment	2016	FPP1	PRJNA750067	SAMN20429684	Chiaverini et al., 2021	7
2016.TE.3350.1.28	Environment	2016	FPP1	PRJNA750067	SAMN20429685	Chiaverini et al., 2021	7
2016.TE.3350.1.40	Environment	2016	FPP1	PRJNA750067	SAMN20429687	Chiaverini et al., 2021	7
2016.TE.3350.1.42	Environment	2016	FPP1	PRJNA750067	SAMN20429688	Chiaverini et al., 2021	7
2016.TE.3350.1.44	Environment	2016	FPP1	PRJNA750067	SAMN20429689	Chiaverini et al., 2021	7

2016.TE.3350.1.50	Environment	2016	FPP1	PRJNA750067	SAMN20429690	Chiaverini et al., 2021	7
2016.TE.3350.1.51	Environment	2016	FPP1	PRJNA750067	SAMN20429691	Chiaverini et al., 2021	7
2016.TE.3350.1.52	Hog head cheese	2016	FPP1	PRJNA750067	SAMN20429692	Chiaverini et al., 2021	7
2016.TE.3350.1.55	Hog head cheese	2016	FPP1	PRJNA750067	SAMN20429693	Chiaverini et al., 2021	7
2016.TE.3350.1.56	Environment	2016	FPP1	PRJNA750067	SAMN20429694	Chiaverini et al., 2021	7
2016.TE.3350.1.57	Environment	2016	FPP1	PRJNA750067	SAMN20429695	Chiaverini et al., 2021	7
2016.TE.3350.1.7	Hog head cheese	2016	FPP1	PRJNA750067	SAMN20429696	Chiaverini et al., 2021	7
2016.TE.3350.1.9	Hog head cheese	2016	FPP1	PRJNA750067	SAMN20429698	Chiaverini et al., 2021	7
2016.TE.5761.1.62	Clinical	2016	NA	PRJNA750067	SAMN20429699	Duranti et al., 2018	7
2016.TE.5761.1.63	Clinical	2016	NA	PRJNA750067	SAMN20429700	Duranti et al., 2018	7
2016.TE.5761.1.64	Clinical	2016	NA	PRJNA750067	SAMN20429701	Duranti et al., 2018	7
2016.TE.5761.1.65	Clinical	2016	NA	PRJNA750067	SAMN20429702	Duranti et al., 2018	7
2016.TE.5761.1.66	Clinical	2016	NA	PRJNA750067	SAMN20429703	Duranti et al., 2018	7
2016.TE.5761.1.67	Clinical	2016	NA	PRJNA750067	SAMN20429704	Duranti et al., 2018	7
2016.TE.5761.1.70	Salami	2016	FPP2	PRJNA750067	SAMN20429706	Chiaverini et al., 2021	7
2016.TE.5761.1.71	Salami	2016	FPP2	PRJNA750067	SAMN20429707	Chiaverini et al., 2021	7
2016.TE.5761.1.73	Salami	2016	FPP2	PRJNA750067	SAMN20429708	Chiaverini et al., 2021	7
2016.TE.5761.1.74	Salami	2016	FPP2	PRJNA750067	SAMN20429709	Chiaverini et al., 2021	7
2016.TE.5761.1.75	Salami	2016	FPP2	PRJNA750067	SAMN20429710	Chiaverini et al., 2021	7
2016.TE.5761.1.76	Salami	2016	FPP2	PRJNA750067	SAMN20429711	Chiaverini et al., 2021	7
2016.TE.6891.1.61	Salami	2016	FPP2	PRJNA750067	SAMN20429712	Chiaverini et al., 2021	7
2016.TE.6891.1.62	Salami	2016	FPP2	PRJNA750067	SAMN20429713	Chiaverini et al., 2021	7
2016.TE.6891.1.63	Salami	2016	FPP2	PRJNA750067	SAMN20429714	Chiaverini et al., 2021	7
2016.TE.6891.1.64	Salami	2016	FPP2	PRJNA750067	SAMN20429715	Chiaverini et al., 2021	7
2016.TE.6891.1.65	Salami	2016	FPP2	PRJNA750067	SAMN20429716	Chiaverini et al., 2021	7
2016.TE.6891.1.66	Salami	2016	FPP2	PRJNA750067	SAMN20429717	Chiaverini et al., 2021	7
2016.TE.6891.1.67	Salami	2016	FPP2	PRJNA750067	SAMN20429718	Chiaverini et al., 2021	7
2016.TE.6891.1.68	Salami	2016	FPP2	PRJNA750067	SAMN20429719	Chiaverini et al., 2021	7
2016.TE.6891.1.75	Clinical	2016	NA	PRJNA750067	SAMN20429721	Chiaverini et al., 2021	7
2016.TE.6891.1.76	Clinical	2016	NA	PRJNA750067	SAMN20429722	Duranti et al., 2018	7
2016.TE.6891.1.79	Environment	2016	RS3	PRJNA750067	SAMN21220764	Chiaverini et al., 2021	7
2016.TE.6891.1.80	Environment	2016	RS3	PRJNA750067	SAMN20429723	Chiaverini et al., 2021	7

2016.TE.6891.1.81	Environment	2016	RS3	PRJNA750067	SAMN20429724	Chiaverini et al., 2021	7
2016.TE.6891.1.82	Environment	2016	RS3	PRJNA750067	SAMN20429725	Chiaverini et al., 2021	7
2016.TE.6891.1.83	Environment	2016	RS3	PRJNA750067	SAMN20429726	Chiaverini et al., 2021	7
2016.TE.6891.1.84	Salami	2016	FPP1	PRJNA750067	SAMN20429727	Chiaverini et al., 2021	7
2016.TE.6891.1.85	Loin	2016	FPP1	PRJNA750067	SAMN20429728	Chiaverini et al., 2021	7
2016.TE.6891.1.86	Salami	2016	FPP1	PRJNA750067	SAMN20429729	Chiaverini et al., 2021	7
2016.TE.6891.1.90	Pork cheeck	2016	FPP1	PRJNA750067	SAMN20429730	Chiaverini et al., 2021	7
2016.TE.6891.1.91	Pork cheeck	2016	FPP1	PRJNA750067	SAMN20429731	Chiaverini et al., 2021	7
2016.TE.6891.1.92	Pork cheeck	2016	FPP1	PRJNA750067	SAMN20429732	Chiaverini et al., 2021	7
2016.TE.6891.1.93	Pork cheeck	2016	FPP1	PRJNA750067	SAMN20429733	Chiaverini et al., 2021	7
2016.TE.6891.1.94	Pork cheeck	2016	FPP1	PRJNA750067	SAMN20429734	Chiaverini et al., 2021	7
2016.TE.7723.1.41	Hog head cheese	2016	FPP1	PRJNA750067	SAMN20429735	Chiaverini et al., 2021	7
2016.TE.7723.1.42	Hog head cheese	2016	FPP1	PRJNA750067	SAMN20429736	Chiaverini et al., 2021	7
2016.TE.7723.1.43	Hog head cheese	2016	FPP1	PRJNA750067	SAMN20429737	Chiaverini et al., 2021	7
2016.TE.7723.1.44	Hog head cheese	2016	FPP1	PRJNA750067	SAMN20429738	Chiaverini et al., 2021	7
2016.TE.7723.1.46	Hog head cheese	2016	FPP1	PRJNA750067	SAMN20429739	Chiaverini et al., 2021	7
2016.TE.7723.1.47	Hog head cheese	2016	FPP1	PRJNA750067	SAMN20429740	Chiaverini et al., 2021	7
2016.TE.7723.1.48	Hog head cheese	2016	FPP1	PRJNA750067	SAMN20429741	Chiaverini et al., 2021	7
2016.TE.7723.1.50	Hog head cheese	2016	FPP1	PRJNA750067	SAMN20429742	Chiaverini et al., 2021	7
2016.TE.7723.1.51	Hog head cheese	2016	FPP1	PRJNA750067	SAMN20429743	Chiaverini et al., 2021	7
2016.TE.7723.1.52	Hog head cheese	2016	FPP1	PRJNA750067	SAMN20429744	Chiaverini et al., 2021	7
2016.TE.7723.1.53	Hog head cheese	2016	FPP1	PRJNA750067	SAMN20429745	Chiaverini et al., 2021	7
2016.TE.7723.1.54	Hog head cheese	2016	FPP1	PRJNA750067	SAMN20429746	Chiaverini et al., 2021	7
2016.TE.8594.1.5	Environment	2016	FPP3	PRJNA750067	SAMN21220765	Chiaverini et al., 2021	7
2016.TE.8594.1.6	Environment	2016	FPP3	PRJNA750067	SAMN20429747	Chiaverini et al., 2021	7
2016.TE.8594.1.7	Environment	2016	FPP3	PRJNA750067	SAMN20429748	Chiaverini et al., 2021	7
2016.TE.8594.1.8	Environment	2016	FPP3	PRJNA750067	SAMN20429749	Chiaverini et al., 2021	7
2016.TE.9198.1.20	Environment	2016	FPP3	PRJNA750067	SAMN20429750	Chiaverini et al., 2021	7
2016.TE.9198.1.21	Environment	2016	FPP3	PRJNA750067	SAMN20429751	Chiaverini et al., 2021	7
2016.TE.9198.1.22	Environment	2016	FPP3	PRJNA750067	SAMN20429752	Chiaverini et al., 2021	7
2016.TE.9198.1.23	Environment	2016	FPP3	PRJNA750067	SAMN20429753	Chiaverini et al., 2021	7
2016.TE.9198.1.26	Environment	2016	FPP3	PRJNA750067	SAMN20429754	Chiaverini et al., 2021	7

2018.TE.5376.1.4	Clinical	2018	NA	PRJNA750067	SAMN20429755	Orsini et al., 2018b (CP029372)	7
2018.TE.5686.1.2	Environment	2016	PH2	PRJNA750067	SAMN20429756	Chiaverini et al., 2021	7
2018.TE.5686.1.3	Environment	2016	PH2	PRJNA750067	SAMN20429757	Chiaverini et al., 2021	7
2018.TE.5686.1.6	Environment	2016	PH2	PRJNA750067	SAMN20429758	Chiaverini et al., 2021	7
2018.TE.6199.1.10	Environment	2016	PH2	PRJNA750067	SAMN20429759	Chiaverini et al., 2021	7
2018.TE.6199.1.11	Environment	2016	PH2	PRJNA750067	SAMN20429760	Chiaverini et al., 2021	7
2018.TE.6199.1.14	Environment	2016	PH2	PRJNA750067	SAMN20429761	Chiaverini et al., 2021	7
2018.TE.6199.1.15	Environment	2016	PH2	PRJNA750067	SAMN20429762	Chiaverini et al., 2021	7
2018.TE.6199.1.16	Environment	2016	PH2	PRJNA750067	SAMN20429763	Chiaverini et al., 2021	7
2018.TE.6199.1.18	Environment	2018	RS5	PRJNA750067	SAMN20429764	Chiaverini et al., 2021	7
2018.TE.6199.1.19	Environment	2018	RS5	PRJNA750067	SAMN20429765	Chiaverini et al., 2021	7
2018.TE.6199.1.2	Environment	2018	PH2	PRJNA750067	SAMN20429766	Chiaverini et al., 2021	7
2018.TE.6199.1.20	Environment	2018	RS5	PRJNA750067	SAMN20429767	Chiaverini et al., 2021	7
2018.TE.6199.1.21	Environment	2018	RS5	PRJNA750067	SAMN20429768	Chiaverini et al., 2021	7
2018.TE.6199.1.22	Environment	2018	RS5	PRJNA750067	SAMN20429769	Chiaverini et al., 2021	7
2018.TE.6199.1.27	Environment	2018	RS5	PRJNA750067	SAMN20429770	Chiaverini et al., 2021	7
2018.TE.6199.1.28	Environment	2018	RS5	PRJNA750067	SAMN20429771	Chiaverini et al., 2021	7
2018.TE.6199.1.3	Environment	2018	PH2	PRJNA750067	SAMN20429772	Chiaverini et al., 2021	7
2018.TE.6199.1.31	Environment	2018	RS5	PRJNA750067	SAMN20429773	Chiaverini et al., 2021	7
2018.TE.6199.1.4	Environment	2018	PH2	PRJNA750067	SAMN20429774	Chiaverini et al., 2021	7
2018.TE.6199.1.5	Environment	2018	PH2	PRJNA750067	SAMN20429775	Chiaverini et al., 2021	7
2018.TE.6199.1.6	Environment	2018	PH2	PRJNA750067	SAMN20429776	Chiaverini et al., 2021	7
2018.TE.6199.1.7	Environment	2018	PH2	PRJNA750067	SAMN20429777	Chiaverini et al., 2021	7
2018.TE.6199.1.8	Environment	2018	PH2	PRJNA750067	SAMN20429778	Chiaverini et al., 2021	7
2018.TE.6199.1.9	Environment	2018	PH2	PRJNA750067	SAMN20429779	Chiaverini et al., 2021	7
2015.TE.29172.1.12	Clinical	2015	NA	PRJNA750067	SAMN20429780	Duranti et al., 2018	7
2016.TE.10956.1.13	Environment	2016	RS4	/	/	/	7
2017.TE.1028.1.3	Clinical	2016	NA	PRJNA750067	SAMN20429781	Chiaverini et al., 2021	7
2015.TE.21231.1.27	Clinical	2014	NA	PRJNA750067	SAMN20429782	Duranti et al., 2018; Orsini et al., 2018a (CP014261)	7

2019.TE.22285.1.5	null	/	/	/	/	/	7
2019.TE.22285.1.6	null	/	/	/	/	/	7
2022.TE.32578.1.2	Sea Turtles	2022	NA	/	/	This study	388
2022.TE.37041.1.2	Sea Turtles	2022	NA	/	/	This study	L.innocua
2022.TE.37093.1.2	Sea Turtles	2022	NA	/	/	This study	L.innocua
2022.TE.37094.1.2	Sea Turtles	2022	NA	/	/	This study	L.innocua
2022.TE.37095.1.2	Sea Turtles	2022	NA	/	/	This study	204
2023.TE.1767.1.2	Sea Turtles	2022	NA	/	/	This study	L.ivanovii
2022.TE.25575.1.2	Sea Turtles	2022	NA	/	/	This study	7
2023.TE.2348.1.2	Sea Turtles	2023	NA	/	/	This study	219
2023.EXT.0907.1659.580	meat environment	2012	NA	PRJNA320339	SAMN04932633	Fox et al., 2016	204
2023.EXT.0907.1655.390	meat	2015	NA	PRJNA320339	SAMN04932632	Fox et al., 2016	204
2023.EXT.0907.1653.510	meat	2015	NA	PRJNA320339	SAMN04932631	Fox et al., 2016	204
2023.EXT.0907.1650.480	dairy environment	2006	NA	PRJNA320339	SAMN04932629	Fox et al., 2016	204
2023.EXT.0907.1651.530	dairy environment	2015	NA	PRJNA320339	SAMN04932630	Fox et al., 2016	204
2023.EXT.0907.1649.340	dairy milk	2012	NA	PRJNA320339	SAMN04932628	Fox et al., 2016	204
2023.EXT.0907.1648.270	dairy cheese	2012	NA	PRJNA320339	SAMN04932627	Fox et al., 2016	204
2023.EXT.0907.1014.080	/	/	NA	PRJEB10780	SAMEA3540881	Institut Pasteur	204
2023.EXT.0907.1229.110	dairy cheese	2012	NA	PRJNA320339	SAMN04932625	Fox et al., 2016	204
2023.EXT.0907.1227.470	dairy cheese	2011	NA	PRJNA320339	SAMN04932624	Fox et al., 2016	204
2023.EXT.0907.1048.320	dairy cheese	2010	NA	PRJNA320339	SAMN04932623	Fox et al., 2016	204
2023.EXT.0907.1016.190	meat ham	2000	NA	PRJNA320339	SAMN04932619	Fox et al., 2016	204
2023.EXT.0907.1017.580	meat environment	2007	NA	PRJNA320339	SAMN04932620	Fox et al., 2016	204
2023.EXT.0907.1019.310	dairy cheese	2009	NA	PRJNA320339	SAMN04932621	Fox et al., 2016	204
2023.EXT.0907.1020.540	dairy cheese	2009	NA	PRJNA320339	SAMN04932622	Fox et al., 2016	204
2023.EXT.0907.1703.270	environmental/food	2016	NA	PRJNA215355	SAMN22866749	Chen et al., 2022	219
2023.EXT.0907.1706.590	environmental/food	2016	NA	PRJNA215355	SAMN22867004	Chen et al., 2022	219
2023.EXT.0907.1710.340	environmental/food	2017	NA	PRJNA215355	SAMN22883519	Chen et al., 2022	219
2023.EXT.0907.1720.030	stool Snapping turtle	2019	NA	PRJNA514286	SAMN13148884	Chen et al., 2022	388
2023.EXT.0907.1716.370	stool Mouse	2019	NA	PRJNA514286	SAMN13148881	Chen et al., 2022	388
