# Supplementary material for: Genomic Characterization of Listeria monocytogenes and Other Listeria Species Isolated from Sea Turtles
Source: Microorganisms. 2024 Apr 18;12(4):817. doi: 10.3390/microorganisms12040817 (PMC11052188; doi:10.3390/microorganisms12040817)
Supplement: Supplementary file 1 [file microorganisms-12-00817-s001.zip › microrganism 2912735_Table S2. Gene presence between sea turtle strains..pdf]

Table S2. Gene presence between sea turtle strains.

2022.TE.32578.1.2	2022.TE.37041.1.2	2022.TE.37093.1.2	2022.TE.37094.1.2	2022.TE.37095.1.2	2023.TE.1767.1.2	2022.TE.25575.1.2	2023.TE.2348.1.2
L. monocytogenes	*L. innocua*	*L. innocua*	*L. innocua*	*L. monocytogenes*	*L. ivanovii*	*L. monocytogenes*	*L.monocytogenes*
ST388	ST542	ST536	ST1481	ST204	NA	ST7	ST219
fosX	*fosX*	*fosX*	*fosX*	*fosX*	*fosX*	*fosX*	*fosX*
lmo0919	*lmo0919*	*lmo0919*	*lmo0919*	*lmo0919*	*lmo0919*	*lmo0919*	*lmo0919*
norB	*norB*	*norB*	*norB*	*norB*	*norB*	*norB*	*norB*
sul	*sul*	*sul*	*sul*	*sul*	*sul*	*sul*	*sul*
lmo169	*lmo169*	*lmo169*	*lmo169*	*lmo169*	*lmo169*	*lmo169*	*lmo169*
LGI-2_LMSO2310	*LGI-2_LMSO2310*	*LGI-2_LMSO2310*	*LGI-2_LMSO2310*	*LGI-2_LMSO2310*	*LGI-2_LMSO2310*	*LGI-2_LMSO2310*	*LGI-2_LMSO2310*
/	/	/	/	LGI-2	*LGI-2_LMOSA2320*	/	/
/	/	/	/	/	LGI-3_LmUB3PA_1705	/	/
/	/	/	/	/	LGI-3_LmUB3PA_1706	/	/
LGI-3_LmUB3PA_1685	/	/	/	/	/	LGI-3_LmUB3PA_1685	/
/	/	/	/	*qacA*	/	/	/
lmo1799	*lmo1799*	*lmo1799*	*lmo1799*	*lmo1799*	*lmo1799*	*lmo1799*	*lmo1799*
lmo1800	*lmo1800*	*lmo1800*	*lmo1800*	*lmo1800*	*lmo1800*	*lmo1800*	*lmo1800*
	x2	*SSI2_lmo0464*<br>*SSI2_lmo0465*<br>*SSI1_lmo0447*	*SSI2_lmo0464*<br>*SSI2_lmo0465*<br>*SSI1_lmo0447*				

/	/	/	/	*SSI1_lmo0444*, *SSI1_lmo0445*, *SSI1_lmo0446* and *SSI1_lmo0448*	/	*SSI1_lmo0444*, *SSI1_lmo0445*, *SSI1_lmo0446* and *SSI1_lmo0448*	/
