# Supplementary material for: Genomic Characterization of Listeria monocytogenes and Other Listeria Species Isolated from Sea Turtles
Source: Microorganisms. 2024 Apr 18;12(4):817. doi: 10.3390/microorganisms12040817 (PMC11052188; doi:10.3390/microorganisms12040817)
Supplement: Supplementary file 1 [file microorganisms-12-00817-s001.zip › microrganism 2912735_Table S3. GWAS CCT..pdf]

Table S3. GWAS CC7.

Gene	Non-unique Gene name	Annotation	Number_pos _present_in	Number_neg _present_in	Number_pos_n ot_present_in	Number_neg_n ot_present_in	Sensi tivity	Specificity	Odds _ratio	Naive_p
**hin_1**	hin_1	DNA-invertase hin	0	182	1	4	0.0	2.15053763 44086025	0.0	0.026737967 914438505
**group _2727**		hypothetical protein	0	182	1	4	0.0	2.15053763 44086025	0.0	0.026737967 914438505
**mco**	mco	Multicopper oxidase mco	0	182	1	4	0.0	2.15053763 44086025	0.0	0.026737967 914438505
**group _2712**		hypothetical protein	0	182	1	4	0.0	2.15053763 44086025	0.0	0.026737967 914438505
**group _2708**		hypothetical protein	0	182	1	4	0.0	2.15053763 44086025	0.0	0.026737967 914438505
**group _2707**		hypothetical protein	0	182	1	4	0.0	2.15053763 44086025	0.0	0.026737967 914438505
**group _2703**		hypothetical protein	0	182	1	4	0.0	2.15053763 44086025	0.0	0.026737967 914438505
**group _2702**		hypothetical protein	0	182	1	4	0.0	2.15053763 44086025	0.0	0.026737967 914438505
**group _2701**		hypothetical protein	0	182	1	4	0.0	2.15053763 44086025	0.0	0.026737967 914438505
**group _2700**		hypothetical protein	0	182	1	4	0.0	2.15053763 44086025	0.0	0.026737967 914438505
**cadA**	cadA	cadmium resistance protein	0	182	1	4	0.0	2.15053763 44086025	0.0	0.026737967 914438505
**group _2692**		hypothetical protein	0	182	1	4	0.0	2.15053763 44086025	0.0	0.026737967 914438505
**hin_2**	hin_2	DNA-invertase hin	0	182	1	4	0.0	2.15053763 44086025	0.0	0.026737967 914438505
**group _2691**		hypothetical protein	0	182	1	4	0.0	2.15053763 44086025	0.0	0.026737967 914438505
**group _2690**		hypothetical protein	0	182	1	4	0.0	2.15053763 44086025	0.0	0.026737967 914438505
**group _2686**		hypothetical protein	0	182	1	4	0.0	2.15053763 44086025	0.0	0.026737967 914438505

**group_987**		hypothetical protein	0	182	1	4	0.0	2.1505376344086025	0.0	0.026737967914438505
**group_2736**		hypothetical protein	0	181	1	5	0.0	2.6881720430107525	0.0	0.0320855614973262
**group_2731**		hypothetical protein	0	181	1	5	0.0	2.6881720430107525	0.0	0.0320855614973262
**group_2730**		hypothetical protein	0	181	1	5	0.0	2.6881720430107525	0.0	0.0320855614973262
**TSFM**	TSFM	Elongation factor Ts mitochondrial	0	181	1	5	0.0	2.6881720430107525	0.0	0.0320855614973262
**group_2726**		hypothetical protein	0	181	1	5	0.0	2.6881720430107525	0.0	0.0320855614973262
**group_2725**		hypothetical protein	0	181	1	5	0.0	2.6881720430107525	0.0	0.0320855614973262
**group_2724**		hypothetical protein	0	181	1	5	0.0	2.6881720430107525	0.0	0.0320855614973262
**group_2723**		hypothetical protein	0	181	1	5	0.0	2.6881720430107525	0.0	0.0320855614973262
**group_2720**		hypothetical protein	0	181	1	5	0.0	2.6881720430107525	0.0	0.0320855614973262
**group_2719**		hypothetical protein	0	181	1	5	0.0	2.6881720430107525	0.0	0.0320855614973262
**group_2717**		hypothetical protein	0	181	1	5	0.0	2.6881720430107525	0.0	0.0320855614973262
**group_2716**		hypothetical protein	0	181	1	5	0.0	2.6881720430107525	0.0	0.0320855614973262
**npr**	npr	NADH peroxidase	0	181	1	5	0.0	2.6881720430107525	0.0	0.0320855614973262
**group_2715**		hypothetical protein	0	181	1	5	0.0	2.6881720430107525	0.0	0.0320855614973262
**group_2713**		IS6 family transposase ISLmo4	0	181	1	5	0.0	2.6881720430107525	0.0	0.0320855614973262
**group_2711**		hypothetical protein	0	181	1	5	0.0	2.6881720430107525	0.0	0.0320855614973262
**group_2710**		IS6 family transposase IS1297	0	181	1	5	0.0	2.6881720430107525	0.0	0.0320855614973262

**group _2709**		hypothetical protein	0	181	1	5	0.0	2.68817204 30107525	0.0	0.032085561 4973262
**group _2706**		hypothetical protein	0	181	1	5	0.0	2.68817204 30107525	0.0	0.032085561 4973262
**group _2705**		Tn3 family transposase	0	181	1	5	0.0	2.68817204 30107525	0.0	0.032085561 4973262
**soj**	soj	Chromosome-partitioning ATPase Soj	0	181	1	5	0.0	2.68817204 30107525	0.0	0.032085561 4973262
**group _2699**		hypothetical protein	0	181	1	5	0.0	2.68817204 30107525	0.0	0.032085561 4973262
**tnpR**	tnpR	Transposon Tn3 resolvase	0	181	1	5	0.0	2.68817204 30107525	0.0	0.032085561 4973262
**group _2697**		hypothetical protein	0	181	1	5	0.0	2.68817204 30107525	0.0	0.032085561 4973262
**group _2696**		hypothetical protein	0	181	1	5	0.0	2.68817204 30107525	0.0	0.032085561 4973262
**group _2695**		hypothetical protein	0	181	1	5	0.0	2.68817204 30107525	0.0	0.032085561 4973262
**group _2694**		hypothetical protein	0	181	1	5	0.0	2.68817204 30107525	0.0	0.032085561 4973262
**group _2693**		hypothetical protein	0	181	1	5	0.0	2.68817204 30107525	0.0	0.032085561 4973262
**group _2688**		hypothetical protein	0	181	1	5	0.0	2.68817204 30107525	0.0	0.032085561 4973262
**group _2687**		IS6 family transposase IS1216E	0	181	1	5	0.0	2.68817204 30107525	0.0	0.032085561 4973262
**group _2735**		hypothetical protein	0	180	1	6	0.0	3.22580645 1612903	0.0	0.037433155 0802139
**group _2734**		hypothetical protein	0	180	1	6	0.0	3.22580645 1612903	0.0	0.037433155 0802139
**group _2733**		hypothetical protein	0	180	1	6	0.0	3.22580645 1612903	0.0	0.037433155 0802139
**group _2732**		IS21 family transposase ISLmo7	0	180	1	6	0.0	3.22580645 1612903	0.0	0.037433155 0802139

**group _2729**		IS3 family transposase ISLmo9	0	180	1	6	0.0	3.225806451612903	0.0	0.0374331550802139
**clpE_2**	clpE_2	ATP-dependent protease	0	180	1	6	0.0	3.225806451612903	0.0	0.0374331550802139
**group _2722**		hypothetical protein	0	180	1	6	0.0	3.225806451612903	0.0	0.0374331550802139
**group _2721**		hypothetical protein	0	180	1	6	0.0	3.225806451612903	0.0	0.0374331550802139
**group _2714**		ISLre2 family transposase ISAot1	0	180	1	6	0.0	3.225806451612903	0.0	0.0374331550802139
**group _2704**		hypothetical protein	0	180	1	6	0.0	3.225806451612903	0.0	0.0374331550802139
**group _2698**		IS3 family transposase ISLmo8	0	180	1	6	0.0	3.225806451612903	0.0	0.0374331550802139
**group _2689**		hypothetical protein	0	180	1	6	0.0	3.225806451612903	0.0	0.0374331550802139
**group _2728**		hypothetical protein	0	179	1	7	0.0	3.763440860215054	0.0	0.0427807486631016
